# Dysregulation of the Immune System in HIV/HCV-Coinfected Patients According to Liver Stiffness Status

**DOI:** 10.3390/cells7110196

**Published:** 2018-11-02

**Authors:** Pilar Garcia-Broncano, Luz Maria Medrano, Juan Berenguer, Juan González-García, Mª Ángeles Jiménez-Sousa, Ana Carrero, Victor Hontañón, Josep M. Guardiola, Manuel Crespo, Carmen Quereda, José Sanz, Ana Belen García-Gómez, Jose Luis Jimenez, Salvador Resino, the GESIDA 3603b Study Group

**Affiliations:** 1 Ragon Institute of MGH, MIT and Harvard, Cambridge, MA 02139, USA; pgarciabroncano@gmail.com; 2 Viral Infection and Immunity Unit, National Center of Microbiology, Health Institute Carlos III, 28220 Madrid, Spain; luzmedranodios@gmail.com (L.M.M.); jimenezsousa@isciii.es (M.Á.J.-S.); piperangarcia@yahoo.es (A.B.G.-G.); 3 Infectious Disease/HIV Unit, Gregorio Marañón G. University Hospital, 28007 Madrid, Spain; jbb4@me.com (J.B.); ana_carrero@yahoo.es (A.C.); 4 Gregorio Marañón Health Research Institute, 28007 Madrid, Spain; joseluis.jimenez@salud.madrid.org; 5 HIV Unit, Internal Medicine Service, La Paz University Hospital, 28046 Madrid, Spain; juangonzalezgar@gmail.com (J.G.-G.); victor.hontanon@gmail.com (V.H.); 6 La Paz Hospital Health Research Institute, 28046 Madrid, Spain; 7 Santa Creu i Sant Pau Hospital, 08041 Barcelona, Spain; jguardiola@santpau.es; 8 Infectious Disease Unit, Internal Medicine Department, Vigo University Hospital Complex, Galicia Sur Health Research Institute, 36312 Vigo, Pontevedra, Spain; manuelcrespocasal@gmail.com; 9 Ramón y Cajal University Hospital, 28034 Madrid, Spain; cqueredar.hrc@salud.madrid.org; 10 Príncipe de Asturias University Hospital, 28805 Madrid, Spain; jsanz.hupa@salud.madrid.org; 11 Laboratory Platform, Gregorio Marañón G. University Hospital, 28007 Madrid, Spain; 12 Bioengineering, Biomaterials and Nanomedicine Networking Biomedical Research Center (CIBER-BBN), Health Institute Carlos III, 28029 Madrid, Spain

**Keywords:** chronic hepatitis C, HIV, cirrhosis, Treg cells, cytokines, immune dysfunction

## Abstract

Background: Advanced cirrhosis is related to alterations in immunity. We aimed to evaluate the levels of peripheral CD4^+^ T cells (Tregs) and plasma cytokine in patients coinfected with human immunodeficiency virus and hepatitis C virus (HIV/HCV) according to liver fibrosis stages [evaluated as liver stiffness measure (LSM)] and their linear relationship. Methods: We performed a cross-sectional study on 238 HIV/HCV-coinfected patients (119 had <12.5 kPa, 73 had 12.5–25 kPa, and 46 had >25 kPa). Peripheral T-cell subsets were phenotyped by flow cytometry, plasma biomarkers were assessed by multiplex immunoassays, and LSM was assessed by transient elastography. Results: We found HIV/HCV-coinfected patients had higher values of CD4^+^ Tregs (*p* < 0.001), memory Tregs (*p* ≤ 0.001), and plasma cytokine levels [IFN-γ (*p* ≤ 0.05) and IL-10 (*p* ≤ 0.01)] compared with healthy donors and HIV-monoinfected patients. In the multivariate analysis, higher LSM values were associated with reduced levels of IL-10 (adjusted arithmetic mean ratio (aAMR) = 0.83; *p* = 0.019), IL-2 (aAMR = 0.78; *p* = 0.017), TNF-α (aAMR = 0.67; *p* < 0.001), and IL-17A (aAMR = 0.75; *p* = 0.006). When we focus on HIV/HCV-coinfected patients analyzed by LSM strata, patients with ≥25 kPa had lower values of IL-2 (aAMR = 0.66; *p* = 0.021), TNF-α (aAMR = 0.565; *p* = 0.003), and IL-17A (aAMR = 0.58; *p* = 0.003) than patients with <12.5 kPa. Conclusion: HIV/HCV-coinfected patients showed an immunosuppressive profile compared to healthy controls and HIV-monoinfected patients. Additionally, HIV/HCV-coinfected patients with advanced cirrhosis (LSM ≥ 25 kPa) had the lowest plasma values of cytokines related to Th1 (IL-2 and TNF-α) and Th17 (IL-17A) response.

## 1. Introduction

Human immunodeficiency virus (HIV) infects CD4^+^ T cells, causing a progressive immunodeficiency in the absence of combination antiretroviral therapy (cART), resulting in the progression to acquired immune deficiency syndrome (AIDS) [1]. These deficits in the immune system are not entirely reversed by suppressive cART, and neither are the effects on CD4^+^ T helper (Th) type 1 (Th1), Th2, Th17, and regulatory CD4^+^ T cell (Tregs) responses [1,2,3,4,5,6]. Th1 cells release interferon IFN gamma (IFN-γ), tumor necrosis factor alpha (TNF-α), and interleukin 2 (IL-2), which mediate immune responses against intracellular pathogens. Th2 cells produce IL-4, IL-5, and IL-13, which mediate the host defense against extracellular parasites [7]. Th17 cells release IL-17, which regulates inflammatory immune responses and is a key regulator of homeostasis and epithelial barrier function [7]. Regulatory CD4^+^ T cells (Tregs) secrete transforming growth factor beta (TGF-β) and IL-10, which regulate self-tolerance and the immune response in infectious diseases, preventing an excessive immune response by suppressive action [8]. This functional alteration of the immune system (deregulation) has been related to gut mucosal barrier dysfunction, dysbiosis, and residual inflammation [9]; persistent immune activation [4]; HIV persistence [10]; and increased risk for tuberculosis [11,12] and pneumococcal colonization [13] in HIV-infected patients on cART.

Hepatitis C virus (HCV) infection becomes chronically persistent in more than 65–75% of patients infected with HCV [14,15]. The progression of chronic hepatitis C (CHC) usually develops slowly, over several decades [14]. However, CHC may be accelerated by the presence of HIV co-infection [16], which promotes an accelerated progression of liver fibrosis and higher rates of cirrhosis, decompensation, and liver failure than patients monoinfected with HCV [17,18,19]. Additionally, CHC increases mortality both associated and not associated with HIV infection [20,21].

During CHC, a delicate balance between a vigorous immune response and unspecific inflammation determines the rate of CHC progression [22]. Thus, the inappropriate immune response leads to the activation and maintenance of liver fibrosis, as well as progression to cirrhosis in HCV-infected patients [23]. Among the elements of the immune response, Th1, Th2, Th17, and Treg cells have relevant roles in progression to cirrhosis in patients with CHC [23,24]. Thus, there has been a reported decrease of cytotoxic function (Th1 and Th17 response) and increase of inhibitory functions (IL-10 and TGF-β) due to the increased level and function of anti-inflammatory Tregs [23,24]. Moreover, in advanced stages of cirrhosis, cirrhosis-associated immune dysfunction (CAID) may appear, which is an acquired alteration of immune function characterized by an increased inflammatory host response and immunosuppression levels [25]. During the CAID, there is dysregulation of liver-localized and peripheral immune cells that is directly related to the severity of liver injury and plays a crucial role in the progression to liver decompensation and acute chronic liver failure (ACLF) [25].

There are previous data about levels of Tregs population and cytokines during CHC infection [22,26]; however, there is little information about levels of Tregs and plasma cytokines related to immune regulation in HIV/HCV-coinfected patients in different stages of liver fibrosis, particularly in advanced stages of cirrhosis. In our study, we aimed to evaluate the levels of CD4^+^ Tregs and cytokine profiles linked to Th1, Th2, Th17, and Tregs cells in the peripheral blood of HIV/HCV-coinfected patients according to the different stages of liver fibrosis [evaluated as liver stiffness measure (LSM)] and their linear relationship.

## 2. Methods

### 2.1. Study Subjects

We carried out a cross-sectional study of 206 patients selected from the cohort of “Grupo de Estudio del SIDA” (GESIDA 3603b study; see Appendix A) enrolled between February 2012 and February 2016 at 14 centers in Spain. The GESIDA 3603b cohort consisted of either anti-HCV therapy-experienced or -naïve patients, who were candidates to receive HCV therapy with peg-IFN-α/ribavirin or peg-IFN-α/ribavirin/direct-acting antivirals (DAAs), as we have previously described [27]. The selection criteria for our study were: (1) detectable plasma HCV-RNA (HCV+) and proviral DNA (HIV-DNA) in peripheral blood cells (HIV+) by the polymerase chain reaction, (2) valid baseline LSM, (3) fresh blood sample to carry out immunological assays, (4) CD4^+^ T cell count ≥200 cells/µL, and (5) stable cART for at least six months or no need for cART according to the guidelines used in the study period. The exclusion criteria were: (1) acute hepatitis C, (2) co-infection with hepatitis B virus, (3) decompensated liver disease or a prior diagnosis of hepatocellular carcinoma, and (4) an HIV-RNA viral load (>50 copies/mL).

In this study, we also analyzed two control groups, which we have previously described [27] (see Table S1): (i) 32 healthy donors negative for HIV, HCV, and hepatitis B virus (HBV) infection; and (ii) 39 HIV-monoinfected patients with CD4^+^ > 500 cells/mm^3^ and an undetectable HIV viral load (normal standard of HIV-infected patients without HCV and HBV infection).

This work was conducted according to the Declaration of Helsinki. The cohort GESIDA 3603b received the approval of the ethics committees of the participating centers on 15 December 2011. Additionally, the study was approved on 10 May 2011 by the Research Ethics Committee of the Instituto de Salud Carlos III. All participants provided written consent prior to enrollment.

### 2.2. Clinical Data

Medical records were used to obtain the most relevant data about demographics, clinical, virological, and laboratory characteristics, and all the information was recorded at each institution using a standard database via an online form, as we have previously described [27].

The LSM was assessed by transient elastography (FibroScan®, Echosens, Paris, France), with results expressed in kilopascals (kPa), and a range from 2.5 to 75 kPa. Trained operators carried out the transient elastography. Representative measurements of liver stiffness were considered reliable when we obtained ten acquisitions with a success rate ≥60% (interquartile range <30%) [28]. From these LSM values, patients were stratified according to LSM cutoffs previously described: <12.5 kPa (non-cirrhosis, [28]), 12.5 to 25 kPa (non-risk of bleeding varices, [29]), and ≥25 kPa (risk of bleeding varices, [29]). We selected the value of LSM ≥25 kPa as a cut-off of variceal hemorrhage, which is a direct consequence of portal hypertension in people with advanced liver cirrhosis.

### 2.3. Flow Cytometry

**Figure 1:**
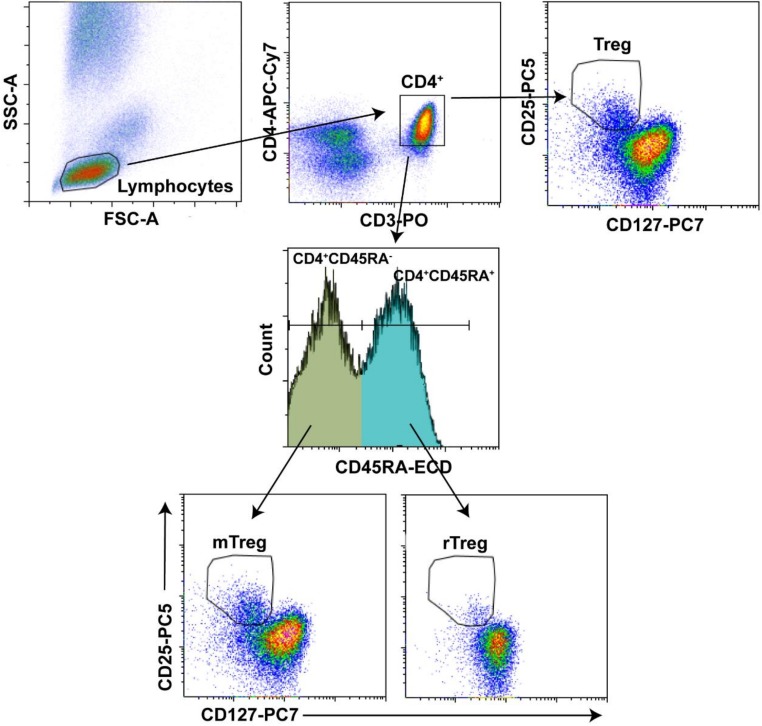
Flow cytometry gating strategy for regulatory T cells subsets. Representative sample of gating strategy used to evaluate the frequency of regulatory T cells subsets is shown. Lymphocytes were first gated on a forward scatter/side scatter (FSC-A/SSC-A) dot plot. The lymphocytes events were visualized using a CD3/CD4 dot plot and the CD4^+^ T cells were gated on a gate CD4^+^. Cells on CD4^+^ are simultaneously displayed on both the CD127/CD25 dot plot and CD45RA histogram. CD127^low/−^CD25^+^ cells appear in gate Treg and subsets CD4^+^CD45RA^−^ and CD4^+^CD45RA^+^ were gated on CD127/CD25 dot plot to visualize mTreg and rTreg cells, respectively.

Tregs may be defined as a subpopulation of CD3^+^CD4^+^CD25^+^CD127^−/low^ T cells that express foxp3 intracellularly [30], and may be subdivided into different subpopulations according to CD45RA expression [activated memory regulatory CD3^+^CD4^+^CD25^+^CD127^−/low^CD45RA^−^ (mTreg) and resting memory regulatory CD3^+^CD4^+^CD25^+^CD127^−/low^CD45RA^+^ (rTreg)] [24]. The expression of CD25, CD127, and CD45RA was evaluated in CD4^+^ T cell subsets by flow cytometry in 100 μL fresh anticoagulated whole blood. The cells were stained with the combination of appropriately titrated antibodies directed to the following surface markers: anti-CD25 PC5 (Phycoerythrin-Cyanin 5.1, clone B1-49.9, Beckman Coulter, Marseille, France), anti-CD127 PC7 (Phycoerythrin-Cyanin 7, clone R34.34, Beckman Coulter, Marseille, France), anti-CD45RA ECD (Phycoerythrin-Texas Red X, clone 2H4LDH11LDB9, Beckman Coulter, Marseille, France), anti-CD4 APC-Cy7 (APC-Cyanine 7, clone OKT4, BioLegend, San Diego, CA, USA), anti-CD8 PB (Pacific Blue, clone SK1, BioLegend, San Diego, CA, USA), and anti-CD3 PO (Pacific Orange, clone VCHT1, Invitrogen, Frederick, MD, USA), and were incubated for 20 min at room temperature in the dark. Next, the IMMUNOPREP Reagent System (Beckman Coulter, Galway, Ireland) was added to each sample using a Coulter MULTI-Q-PREP Lysing Workstation (Beckman Coulter, Miami, FL, USA) to lyse and fixate them. Fluorescence was measured with a Gallios™ flow cytometer (Beckman Coulter, Miami, FL, USA). The number of minimum events was 200,000 cells in the lymphocyte gate for each sample, and flow cytometry data were analyzed using the Kaluza™ software package (version 1.5; Beckman Coulter, Miami, FL, USA). The flow cytometry gating strategy for regulatory T cells subsets is shown in Figure 1.

### 2.4. Multiplex Assay and ELISA

Plasma samples were collected in the Spanish HIV BioBank of Gregorio Marañón University Hospital and stored until use at –80 °C. Plasma cytokines (IFN-γ, IL-12p70, IL-17A, IL-2, IL-4, IL-10, and TNF-α) were measured by the ProcartaPlex^TM^ multiplex immunoassay (Bender MedSystems GmbH, Vienna, Austria) using a Luminex 200™ analyzer (Luminex Corporation, Austin, TX, USA). A specific commercial ELISA test was used to measure plasma TGF-β1 levels according to the manufacturer’s procedure (Bender MedSystems GmbH, Vienna, Austria).

### 2.5. Statistical Analysis

The statistical analysis was performed with the Statistical Package for the Social Sciences (SPSS) 21.0 (SPSS INC, Chicago, IL, USA). Statistical significance was defined as *p* < 0.05. All *p*-values were two-tailed.

For the descriptive study, values were expressed as an absolute number (percentage) and median (25th; 75th percentile). Categorical data and proportions were analyzed using the chi-squared test or Fisher’s exact test, as required. Kruskal-Wallis and Mann-Whitney tests were used to compare data among independent groups.

We also used Generalized Linear Models (GLM), with a gamma distribution (log-link), for evaluating the adjusted association between LSM values and levels of biomarkers in peripheral blood. This test gives the arithmetic mean ratio (AMR) or the value by which the arithmetic mean of the primary outcome is multiplied. Each regression test was adjusted by age, gender, baseline CD4^+^ T cells, HIV viral load (≥50 cp/mL), diabetes, high alcohol intake, previous IFNα HCV therapy, log_10_ HCV RNA, HCV-GT1, and prior AIDS diagnosis.

## 3. Results

### 3.1. Patients

Table S1 shows the characteristics of the subjects included in this study. HIV/HCV-coinfected patients had the highest frequencies of males, alcohol ex-drinker, HIV acquired by IVDU, antiretroviral therapy with 2NRTI+II-based or 2NRTI+PI-based, Nadir CD4^+^ T-cells < 200 cells/mm^3^, and CD4^+^ T-cells < 500 cells/mm^3^. 

The characteristics of HIV/HCV-coinfected patients stratified by LSM are shown in Table 1. We only found significant differences among groups in the percentages of patients with previous peg-IFNα HCV therapy (*p* = 0.017), absolute count of CD4^+^ T cells (*p* = 0.023), and HCV-GT4 (*p* = 0.027).

### 3.2. Biomarker Values in HIV/HCV-Coinfected Patients and Control Groups: Univariate Analysis

Overall, HIV/HCV-coinfected patients showed significantly higher proportions of CD3^+^CD4^+^CD25^+^CD127^−/low^ (Treg) (*p* ≤ 0.001), CD3^+^CD4^+^CD25^+^CD127^−/low^CD45RA^−^ (mTreg) (*p* ≤ 0.001), and plasma cytokine levels [IFN-γ (*p* ≤ 0.05) and IL-10 (*p* ≤ 0.01)] compared with healthy controls and HIV-monoinfected patients (Table 2).

We subsequently analyzed HIV/HCV-coinfected patients according to LSM stratification (Table 3). Patients with LSM 12.5–25 kPa had lower values of IL-12p70 (*p* = 0.017), TNF-α (*p* = 0.043), IL-4 (*p* = 0.032), and IL-17A (*p* = 0.027) than patients with LSM <12.5 kPa. Additionally, patients with LSM ≥ 25 kPa had reduced values of IL-2 (*p* = 0.039), TNF-α (*p* = 0.003), IL-4 (*p* = 0.038), and IL-17A (*p* = 0.003) than patients with LSM < 12.5 kPa. In contrast, patients with LSM ≥ 25 kPa had higher values of IFN-γ (*p* = 0.041) than patients with LSM 12.5–25 kPa.

### 3.3. Association between Biomarker Values and Liver Stiffness: Multivariate Analysis

**Figure 2:**
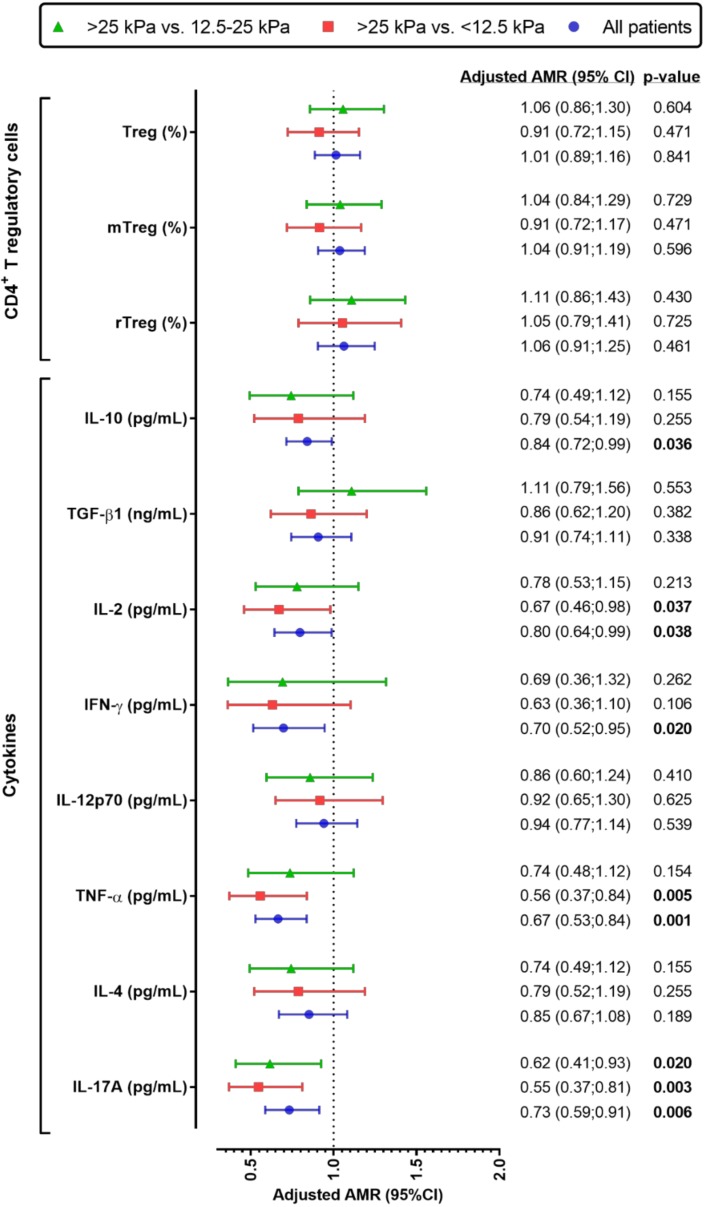
Values of the relationship between liver stiffness measure (continuous and categorical) and peripheral blood biomarkers (CD4^+^ Treg cells and plasma cytokines), adjusted by the main clinical and epidemiological covariables, in HIV/HCV-coinfected patients. Blue (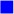): Values of adjusted arithmetic mean ratio (aAMR) in HIV/HCV-coinfected patients. Red (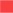): Values of aAMR for patients with >25 kPa versus patients with <12.5 kPa (reference). Green (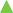): Values of aAMR for patients with >25 kPa versus patients with 12.5–25 kPa (reference). Statistics. *p*-values were calculated by the Generalized Linear Models test with a gamma distribution (log-link). Each regression test was adjusted by age, gender, baseline CD4^+^ T cells, HIV viral load (≥50 cp/mL), high alcohol intake, diabetes, log_10_ HCV RNA, HCV-GT1, previous IFNα HCV therapy, and prior AIDS diagnosis. Abbreviations: HCV, hepatitis C virus; HIV, human immunodeficiency virus; LSM, liver stiffness measure; aAMR, adjusted arithmetic mean ratio; CI, confidence interval; CDXX, cluster of differentiation; TGF-β1, transforming growth factor beta 1; IFN-γ, Interferon gamma; IL-XX, interleukin; Treg cells, regulatory CD4^+^ T cells; TNF-α, tumor necrosis factor alpha.

We examined the relationship of LSM values (continuous variable) with biomarkers of CD4^+^ Treg cells and plasma cytokines (Figure 2). We found that higher LSM values were independently associated with lower plasma levels of IL-10 (aAMR = 0.84; *p* = 0.038), IL-2 (aAMR = 0.80; *p* = 0.038), IFN-γ (aAMR = 0.70; *p* = 0.020), TNF-α (aAMR = 0.67; *p* = 0.001), and IL-17A (aAMR = 0.73; *p* = 0.006). When we focus on HIV/HCV-coinfected patients analyzed by LSM strata (ordinal variable), patients with ≥25 kPa (reference group) had lower plasma values of IL-2 (aAMR = 0.67; *p* = 0.037), TNF-α (aAMR = 0.56; *p* = 0.005), and IL-17A (aAMR = 0.55; *p* = 0.003) than patients with <12.5 kPa; and IL-17A (aAMR = 0.62; *p* = 0.020) than patients with 12.5–25 kPa.

## 4. Discussion

In this study, we found that HIV/HCV-coinfected patients had higher percentages of peripheral Tregs [(CD3^+^CD4^+^CD25^+^CD127^−/low^) and mTregs (CD3^+^CD4^+^CD25^+^CD127^−/low^CD45RA^−^)] and plasma cytokine levels (IFN-γ and IL-10) than healthy controls or HIV-monoinfected patients. Moreover, HIV/HCV-coinfected patients showed an inverse relationship between LSM values (continuous variable) and plasma cytokines [Treg (IL-10), Th1 (IL-2, IFN-γ, and TNF-α), and Th17 (IL-17A)]. Additionally, patients with cirrhosis who had LSM ≥25 kPa showed the lowest values of plasma cytokines [Th1 (IL-2 and TNF-α) and Th17 (IL-17A)]. It should be noted that the differences found among groups in plasma cytokine levels are small, but these differences have to be interpreted taking into account the range of plasma values detected in our assay, which are similar to those found by other authors in plasma or serum [31,32,33]. 

Moreover, we have also stratified our HIV/HCV coinfected patients by other LSM cut-off points, such as <7.1 kPa (F0–F1; minimal fibrosis), 7.1–9.4 kPa (F2; moderate fibrosis), 9.5–12.4 kPa (F3; advanced fibrosis), and ≥12.5 kPa (F4; cirrhosis) [34]. However, no association was found among these LSM stages (F0 to F4, and F0-F3 vs. F4) and the studied biomarkers in our patients (data not shown). Therefore, we showed for the first time an association between greater liver stiffness and increased dysregulation of the immune system in HIV/HCV-coinfected patients with compensated cirrhosis, an alteration which becomes more evident in patients with advanced cirrhosis [≥25 kPa (risk of bleeding varices)]. It is possible that this secondary immunosuppression may be mobilized to counteract the chronic immune activation due to the microbial translocation we described in a recent article [27], resulting in sustained activation of the immunosuppressor machinery of the host [25].

CD4^+^ Treg cells lead to marked deregulation and suppression of the immune system during HIV and HCV infections, promoting progression to AIDS, the development of fibrosis and cirrhosis, and their persistence [8]. In our study, patients mono-infected with HIV had higher percentages of CD4^+^ Treg cells than healthy donors, and HIV/HCV-coinfected patients had higher percentages of Tregs and mTregs than healthy controls or HIV-monoinfected patients. However, we did not find any significant differences in rTregs among groups, so the differences in Tregs were restricted to the mTreg subset. During a primary immune response, antigen-presenting cells (ACP) activated rTregs by presenting antigens and providing co-stimulatory signals, promoting the expansion and functional differentiation of rTregs to effector Tregs and posterior mTreg cells with a potential for long-term survival [35]. Later, during chronic viral infections, mTregs exhibit a peripheral clonal expansion to regulate memory effector responses and thwart collateral damage to tissues [8,35].

Previous reports have shown higher frequencies of circulating Tregs in HCV-monoinfected patients [36,37,38] and HIV-monoinfected patients [39,40,41] compared to healthy donors. However, Treg data from HIV/HCV-coinfected patients described in the literature are inconsistent. Rallon et al. showed no significant differences in circulating Tregs (CD4^+^CD25^+^FoxP3^+^) among HIV/HCV-coinfected patients, HIV-monoinfected patients, and heathy controls, but higher levels than HCV-monoinfected patients [41]. Cho et al. showed higher values of Tregs (CD4^+^FoxP3^+^) in HIV/HCV-coinfected patients than HCV-monoinfected patients, HIV-monoinfected patients, and seronegative controls [42]. Hartling et al. found higher values of Tregs (CD4^+^CD25^+^CD127^low^Foxp3^+^) in HIV/HCV-coinfected patients than HCV-monoinfected patients and seronegative controls [43]. These three articles had a lower number of patients in each study group than our study and their patients infected with HIV had lower CD4^+^ T-cell counts and a higher percentage of detectable HIV viral load [41,42,43]. Moreover, we found that HIV/HCV-coinfected patients did not have any significant relationship between LSM values and the frequencies of Treg subsets in peripheral blood—data consistent with previous reports [42,43,44]. In this regard, it has been described that neither of the DAA regimens, with and without IFNα, have been able to normalize the frequencies and the activation of Tregs one year after HCV elimination [45], which could contribute to the development of immune dysfunction, and non-AIDS- and AIDS-related complications in HIV/HCV-coinfected patients.

Moreover, CD4^+^ Treg cells secrete IL-10 and TGF-β1, which regulate the immune system and the response against pathogens [8]. On the one hand, IL-10 inhibits the synthesis of proinflammatory cytokines (TNFα, IL-1β), Th1 cytokines (IL-2, IL-12, IFNγ), Th2 cytokines (IL-4, IL-5 and IL-10), and Th17 cytokines (IL-17) [7]. Thus, IL-10 generates a suppressive effect, preventing exacerbations of the immune response and subsequent tissue damage, but it may also facilitate the persistence of chronic viral infections, such as HIV and HCV [46]. CHC patients show higher plasma IL-10 levels than healthy subjects [47,48] or HIV-monoinfected patients [49]. Additionally, increased IL-10 levels have been related to the progression of HIV and HCV infections [50]. On the other hand, TGF-β1 is a cytokine with an immunosuppressive and profibrotic effect [51]. TGF-β1 mediates the suppression of both innate and adaptive immune system cells by blocking the production of TNF-α, IFN-γ, IL-2, IL-4, and IL-12 [51]. The chronic over-production of TGF-β1 has been described as a significant cause of immunosuppression in HIV infection [51]. Also, TGF-β1 promotes the activation of hepatic stellate cells, the accumulation of fibrillar components, progressive fibrosis and cirrhosis, and the development of hepatocellular carcinoma [52]. Here, we show that HIV/HCV-coinfected patients had higher plasma IL-10 values compared to healthy donors and HIV-monoinfected patients, but not in the case of TGF-β1. However, we did not find any significant differences among liver stiffness strata for both cytokines (IL-10 and TGF-β1), which together with similar values of Tregs, could be attributed to the fact that HIV/HCV patients had CD4^+^ T cell counts higher than 200 cells/µL; since significant increases in Treg percentages (and levels of IL-10 and TGF-β1) are preferentially found in patients with CD4^+^ T cell counts below 200 cells/μL [51]. Additionally, the differences in CD4^+^ T cell counts are not very relevant to liver stiffness, and baseline CD4^+^ T cell counts adjusted the statistical analysis in our study.

In our study, HIV/HCV-coinfected patients had an inverse relationship of LSM values and LSM strata with plasma cytokines Th1 (IL-2 and TNF-α) and Th17 (IL-17A) in the multivariate analyses. IL-2 is a key cytokine for the proper functioning of the immune system [53]. Plasma levels of IL-2 decrease both as HIV infection [54] and CHC [55] progress and are associated with poor clinical prognoses in both infections [33,54,56]. TNF-α is a proinflammatory cytokine that regulates the immune system and promotes a response capable of eradicating infectious agents, but can also lead to local injury at the site of infection and harmful systemic effects [57]. TNF-α plays an important role in the pathogenesis of both HIV and HCV infections, and it has been related to AIDS progression and the development of cirrhosis [58,59]. IL-17A promotes a proinflammatory and profibrotic environment in response to chronic viral hepatitis, triggering more tissue injury and dysfunctional reparative responses [23]. However, the destruction of Th17 cells during HIV infection may alter the production of IL-17 and its plasma levels decrease with AIDS progression [60]. As we have commented previously, HIV/HCV-coinfected patients with more advanced cirrhosis have shown higher levels of plasma lipopolysaccharide, a marker of bacterial translocation [27]. Thus, it is possible that patients with LSM ≥25kPa had a lower production of IL-2, TNF-α, and IL-17A due to the taxing effect of bacterial translocation on the immune system, which may induce immune dysfunction and a lower production of key cytokines during severe cirrhosis [25]. Additionally, the absence of significant differences between HIV/HCV-coinfected patients and the control groups may be due to characteristics of the HIV/HCV-coinfected group, which was made up of around 50% cirrhotic patients, and around 20% cirrhotic with LSM ≥25kPa, who showed lower values of IL-2, TNF-α, and IL-17A.

Finally, we must emphasize that we evaluated several subsets of Treg cells and a set of systemic cytokines related to the immune response of Th1, Th2, Th17, and Tregs cells in peripheral blood, which is not the same as measuring these biomarkers in the liver or lymph nodes of HIV/HCV-coinfected patients. However, the detection of biomarkers in peripheral blood (liquid biopsy) can be an alternative to biopsy tissues that are difficult to access [61,62]. Moreover, as mentioned above, HIV infection causes a poor immune function due to CD4^+^ T-cells infection [1], and both HIV and HCV infections promote an immune response to try to control both viral infections [1,22]. Nevertheless, it can also result in a broad and non-specific immune activation that leads to a dysregulated immune function and a variety of tissue injuries, such as accelerated liver fibrosis and other disorders related and non-related to both HCV and HIV infections [8,55,63]. Therefore, the altered biomarkers that we found in our HIV/HCV-coinfected patients may be a consequence of both viral infections; while these altered values of Treg cells and cytokines might also injure the immune system itself, triggering several comorbidities (autoimmune diseases and lymphoproliferative disorders), and damage tissues directly related to both infections (lymph nodes and liver) and other tissues not directly related (cardiovascular, kidney, bones) [64].

## 5. Limitations of Study

Firstly, we used a cross-sectional design with a limited number of patients in some of the study groups, which may entail a lack of uniformity and could limit the possibility of finding significance. Secondly, patients included in this study met a set of criteria for starting HCV treatment (see patients section), which may have introduced a selection bias. Thirdly, a group of HCV-monoinfected patients and patients with decompensated cirrhosis were not used to provide comparator information for our HIV/HCV-coinfected patients with compensated cirrhosis. Fourthly, we did not adjust our results by multiple comparisons. In this regard, we carried out a clinical-orientated study, not a random search of a meaningful result, since our hypothesis is supported by theory and previous reports, and the analyzed biomarkers cannot be considered entirely independent. In these cases, it is not recommended to adjust the “*p*-value” following multiple tests because it can significantly penalize relevant results [65,66]. Fifthly, our study was performed in HIV/HCV-coinfected patients, and it would be interesting to analyze a group of HCV-monoinfected patients to determine whether the increase in CD4^+^ Tregs and cytokine changes in HIV/HCV-coinfected patients are the result of HCV infection, liver disease, or HIV coinfection. However, we did not have access to a cohort of HCV-monoinfected patients. Sixthly, we have not used a fixable Live/Dead dye in our fresh whole blood samples, which may influence the results of flow cytometry. However, it is unlikely that there was a bias with respect to a group, since all the samples were processed in the same way.

## 6. Conclusions

In conclusion, HIV/HCV-coinfected patients showed an immunosuppressive profile compared to healthy controls and HIV-monoinfected patients. Additionally, HIV/HCV-coinfected patients with advanced cirrhosis (LSM ≥25 kPa) had the lowest plasma values of cytokines related to Th1 (IL-2 and TNF-α) and Th17 (IL-17A) response. Our findings could have a relevant role in the assessment of immune function in patients coinfected with HIV/HCV with compensated cirrhosis.

## Figures and Tables

**Table 1:** Clinical and epidemiological characteristics of HIV/HCV-coinfected patients stratified by values of LSM.

Characteristic	<12.5 kPa	12.5–25 kPa	>25 kPa	*p*
No.	102	65	39	-
Age (years)	48 (45; 52)	49 (46; 51)	50 (46; 53)	0.287
Gender (male)	83 (81.4%)	47 (72.3%)	32 (82.1%)	0.320
BMI (kg/m^2^)	23.8 (21.4; 26.1)	25.1 (22.7; 28.1)	24.5 (21.8; 26.5)	0.077
Diabetes	8 (7.8%)	5 (7.7%)	5 (12.8%)	0.605
Current alcohol drinker (>50 g/day)	2 (2%)	3 (4.6%)	1 (2.6%)	0.603
Alcohol ex-drinker	47 (46.1%)	31 (47.7%)	23 (59%)	0.399
HIV acquired by IVDU	79 (77.5%)	49 (77.4%)	32(82.1%)	0.570
Prior AIDS	21 (20.6%)	20 (30.8%)	13 (34.2%)	0.276
Years since HIV diagnosis	23 (17; 26)	24 (20; 26)	21 (17; 25)	0.098
Years since HCV infection	22 (16; 24)	21 (18; 26)	18 (17; 22)	0.170
Previous IFNα HCV-therapy	33 (32.4%)	46 (70.8%)	19 (48.7%)	**0.001**
Antiretroviral therapy				
Non-treated	1 (1%)	0 (0%)	2 (5.3%)	0.122
PI-based	15 (14.7%)	11 (16.9%)	3 (7.7%)	0.432
2NRTI+II-based	28 (27.5%)	17 (26.2%)	8 (20.5%)	0.709
2NRTI+PI-based	23 (22.5%)	9 (13.8%)	10 (26.3%)	0.233
2NRTI+NNRTI-based	29 (28.4%)	23 (35.4%)	12 (31.6%)	0.689
Others	6 (5.9%)	5 (7.7%)	4 (10.2%)	0.523
HIV markers				
Nadir CD4^+^ T cells	198 (99; 277)	162 (83; 234)	167 (84; 242)	0.197
Nadir CD4^+^ T cells < 200 cells/mm^3^	49 (48%)	39 (60%)	25 (64.1%)	0.128
Baseline CD4^+^ T cells	626 (436; 845)	511 (344; 730)	506 (360; 803)	**0.023**
Baseline CD4^+^ T cells < 500 cells/mm^3^	33 (32.4%)	31 (48.4%)	19 (48.7%)	0.062
HCV markers				
HCV genotype				
1	69 (67.6%)	52 (80%)	28 (71.8%)	0.158
2	3 (2.9%)	1 (1.5%)	1 (2.6%)	0.853
3	16 (15.7%)	11 (16.9%)	8 (20.5%)	0. 764
4	14 (13.7%)	1 (1.5%)	2 (5.1%)	**0.016**
Log_10_ HCV-RNA (IU/mL)	6.30 (5.81; 6.80)	6.30 (6.00 6.68)	6.11 (5.74; 6.56)	0.472
HCV-RNA > 850,000 IU/mL	77 (75.5%)	55 (84.6%)	30 (76.9%)	0.251

Statistics: Values expressed as absolute number (percentage) and median (interquartile range). *p*-values were calculated by Chi-square tests and Mann-Whitney tests in HIV/HCV-coinfected patients stratified by LSM (<12.5 kPa, 12.5–25 kPa, and >25 kPa). Abbreviations: LSM, liver stiffness measure; kPa, kilopascals; BMI, body mass index; HCV, hepatitis C virus; HCV-RNA, HCV plasma viral load; HIV-1, human immunodeficiency virus type 1; LSM, liver stiffness measure; HIV-RNA, HIV plasma viral load; IVDU, intravenous drug user; AIDS, acquired immune deficiency syndrome; IFNα+rib, interferon-alpha plus ribavirin; NNRTI, non-nucleoside analogue HIV reverse transcriptase inhibitor; NRTI, nucleoside analogue HIV reverse transcriptase inhibitor; PI, protease inhibitor; II, integrase inhibitor.

**Table 2:** Summary of markers of peripheral CD4^+^ Treg cells and plasma cytokines in healthy donors, HIV-monoinfected patients, and HIV/HCV-coinfected patients.

Biomarkers	Healthy Controls (0)	HIV-mono (1)	HIV/HCV-co (2)	*p* (0–1)	*p* (0–2)	*p* (1–2)
CD4^+^ T regulatory cells (%)						
CD3^+^CD4^+^CD25^+^CD127^−/low^ (Treg)	4.5 (3.3; 5.4)	6.1 (4.9; 7.5)	8.1 (6.3; 10)	**<0.001**	**<0.001**	**<0.001**
CD3^+^CD4^+^CD25^+^CD127^−/low^CD45RA^−^ (mTreg)	5.9 (4.1; 8.2)	7.9 (6.4; 10.8)	10.7 (8.1; 13.8)	**0.001**	**<0.001**	**<0.001**
CD3^+^CD4^+^CD25^+^CD127^−/low^CD45RA^+^ (rTreg)	3 (2.1; 3.8)	3.2 (1.8; 5.5)	3 (2; 4.3)	0.472	0.692	0.524
Cytokines						
IL-10 (pg/mL)	0.5 (0.4; 1.5)	0.8 (0.4; 1.7)	1.4 (0.8; 2.6)	0.533	**0.001**	**0.003**
TGF-β1 (ng/mL)	35.6 (21.2; 53.7)	31.2 (17.7; 51.9)	28.2 (14; 56.9)	0.825	0.382	0.468
IL-2 (pg/mL)	3.6 (1; 4.1)	1.5 (1; 3.7)	3.6 (0.9; 6.5)	0.355	0.778	0.695
IFN-γ (pg/mL)	4.8 (3.5; 8.5)	5.1 (2.8; 9.1)	8.6 (2.6; 26.7)	0.984	**0.048**	**0.014**
IL-12p70 (pg/mL)	1.7 (0.9; 3.2)	1.6 (1.1; 2.5)	1.9 (1.2; 3.9)	0.802	0.390	0.174
TNF-α (pg/mL)	1.5 (0.7; 3.5)	1.6 (0.7; 3)	2.1 (0.9; 5)	0.881	0.167	0.177
IL-4 (pg/mL)	3 (1.4; 6.1)	3.1 (1.8; 5.1)	3 (1.7; 6.8)	0.807	0.497	0.823
IL-17A (pg/mL)	0.9 (0.5; 2.1)	1.5 (1; 2.1)	1.1 (0.5; 2.6)	0.150	0.681	0.233

Statistics: Values expressed as median (interquartile range). *p*-values were calculated by the Mann-Whitney test. Abbreviations: HCV, hepatitis C virus; HIV, human immunodeficiency virus; CDXX, cluster of differentiation; TGF-β1, transforming growth factor beta 1; IFN-γ, Interferon gamma; IL-XX, interleukin; Treg, regulatory CD4^+^ T cells; TNF-α, tumor necrosis factor alpha.

**Table 3:** Summary of markers of peripheral CD4^+^ Treg cells and plasma cytokines in HIV/HCV-coinfected patients according to fibrosis/cirrhosis stage.

Parameter	LSM < 12.5 kpa (0)	LSM 12.5–25 kpa (1)	LSM ≥ 25 Kpa (2)	*p* (0–1)	*p* (0–2)	*p* (1–2)
CD4^+^ T regulatory cells (%)						
CD3^+^CD4^+^CD25^+^CD127^−/low^ (Treg)	8 (6.1; 10)	8.5 (6.2; 10.8)	7.7 (6.4; 9.7)	0.573	0.549	0.327
CD3^+^CD4^+^CD25^+^CD127^−/low^CD45RA^−^ (mTreg)	11.1 (8.5; 13.7)	11.1 (8.1; 14.2)	9.8 (7.6; 12.8)	0.965	0.095	0.207
CD3^+^CD4^+^CD25^+^CD127^−/low^CD45RA^+^ (rTreg)	2.9 (2.2; 3.9)	2.9 (1.7; 5.2)	3.7 (2.5; 4.4)	0.837	0.112	0.491
Cytokines						
IL-10 (pg/mL)	1.5 (0.9; 3)	1.2 (0.7; 2.2)	1.4 (0.7; 2.6)	0.076	0.410	0.383
TGF-β1 (ng/mL)	33 (15.8; 56)	23.5 (12.2; 59.8)	22.3 (10.5; 48.5)	0.275	0.137	0.750
IL-2 (pg/mL)	3.6 (0.9; 7.9)	1.5 (0.9; 5.7)	2.7 (0.9; 3.6)	0.078	**0.039**	0.800
IFN-γ (pg/mL)	10.5 (2.7; 34.5)	6.6 (1.5; 15.2)	12.4 (4.7; 28.3)	0.071	0.833	**0.041**
IL-12p70 (pg/mL)	2.3 (1.4; 4.3)	1.6 (0.9; 3)	1.8 (1.1; 4)	**0.017**	0.227	0.492
TNF-α (pg/mL)	3 (1.3; 6.2)	1.7 (0.9; 4.9)	1.1 (0.4; 3.2)	**0.043**	**0.003**	0.341
IL-4 (pg/mL)	3.8 (1.7; 7.4)	2.4 (1.5; 6)	1.8 (1.1; 6.7)	**0.032**	**0.038**	0.886
IL-17A (pg/mL)	1.7 (0.8; 3.8)	0.9 (0.3; 2)	0.7 (0.4; 2)	**0.008**	**0.003**	0.620

Statistics: Values expressed as median (interquartile range). *P*-values were calculated by the Mann-Whitney test in HIV/HCV-coinfected patients stratified by LSM (<12.5 kPa, 12.5–25 kPa, and >25 kPa). Abbreviations: kPa, kilopascals; HCV, hepatitis C virus; HIV, human immunodeficiency; LSM, liver stiffness measure; CDXX, cluster of differentiation; TGF-β1, transforming growth factor beta 1; IFN-γ, Interferon gamma; IL-XX, interleukin; Treg cells, regulatory CD4^+^ T cells; TNF-α, tumor necrosis factor alpha.

## Supplementary Materials

The following are available online http://www.mdpi.com/2073-4409/7/11/196/s1, Table S1: Characteristics of control groups and HIV/HCV co-infected patients.

## Appendix A

***The GESIDA 3603b Cohort Study Group***

***Hospital General Universitario Gregorio Marañón, Madrid:*** A Carrero, P Miralles, JC López, F Parras, B Padilla, T Aldamiz-Echevarría, F Tejerina, C Díez, L Pérez-Latorre, C Fanciulli, I Gutiérrez, M Ramírez, S Carretero, JM Bellón, J Bermejo, and J Berenguer.

***Hospital Universitario La Paz, Madrid:*** V Hontañón, JR Arribas, ML Montes, I Bernardino, JF Pascual, F Zamora, JM Peña, F Arnalich, M Díaz, J González-García.

***Hospital de la Santa Creu i Sant Pau, Barcelona:*** P Domingo, JM Guardiola.

***Hospital Universitari Vall d’Hebron, Barcelona:*** E Van den Eynde, M Pérez, E Ribera, M Crespo.

***Hospital Universitario Ramón y Cajal, Madrid:*** JL Casado, F Dronda, A Moreno, MJ Pérez-Elías, MA Sanfrutos, S Moreno, C Quereda.

***Hospital Universitario Príncipe de Asturias, Alcalá de Henares:*** A Arranz, E Casas, J de Miguel, S Schroeder, J Sanz.

***Hospital Universitario de La Princesa, Madrid:*** J Sanz, I Santos.

***Hospital Donostia, San Sebastián:*** MJ Bustinduy, JA Iribarren, F Rodríguez-Arrondo, MA Von-Wichmann.

***Hospital Clínico San Carlos, Madrid:*** J Vergas, MJ Téllez.

***Hospital Universitario San Cecilio, Granada:*** D. Vinuesa, L. Muñoz, and J. Hernández-Quero.

***Hospital Clínico Universitario, Valencia:*** A Ferrer, MJ Galindo.

***Hospital General Universitario, Valencia:*** L Ortiz, E Ortega.

***Hospital Universitari La Fe, Valencia:*** M Montero, M Blanes, S Cuellar, J Lacruz, M Salavert, J López-Aldeguer.

***Hospital Universitario de Getafe, Getafe:*** G Pérez, G Gaspar.

***Fundación SEIMC-GESIDA, Madrid:*** M Yllescas, P Crespo, E Aznar, H Esteban
