# Supplementary material for: Dysregulation of the Immune System in HIV/HCV-Coinfected Patients According to Liver Stiffness Status
Source: Cells. 2018 Nov 2;7(11):196. doi: 10.3390/cells7110196 (PMC6262386; doi:10.3390/cells7110196)
Supplement: Supplementary file 1 [file cells-07-00196-s001.pdf]

Table S1. Characteristics of control groups and HIV/HCV co-infected patients.

	**Healthy blood subjects**	**HIV monoinfected patients**	**HIV/HCV coinfected patients**	**p-values**
**No.**	32	39	206	-
**Gender (male)**	17 (53.1%)	24 (61.5%)	162 (78.6%)	**0.002**
**Age (years)**	49.5 (47; 53)	51 (46; 53)	49 (46; 52)	0.342
**BMI (kg/m²)**	24.9 (23.1; 27.1)	25.3 (23.5; 26.6)	24.4 (21.8; 26.8)	0.236
**Diabetes**	-	6 (15.8%)	18 (8.7%)	0.181
**Current alcohol drinker (>50 g/day)**	-	1 (2.5%)	6 (2.9%)	0.891
**Alcohol ex-drinker**	-	0 (0%)	101 (49.3%)	**<0.001**
**HIV acquired by IVDU**	-	0 (0%)	160 (78.8%)	**<0.001**
**Prior AIDS**	-	13 (33.3%)	54 (26.6%)	0.397
**Years since HIV infection**	-	-	23 (18; 26)	-
**Years since HCV infection**	-	-	21 (17; 24)	-
**Antiretroviral therapy**				
**Non treated**	-	0 (0%)	3 (1.5%)	0.446
**PI-based**	-	10 (25.6%)	29 (14.3%)	0.087
**2NRTI+II-based**	-	4 (10.2%)	53 (26.1%)	**0.038**
**2NRTI+PI-based**	-	0 (0%)	42 (20.7%)	**0.003**
**2NRTI+NNRTI-based**	-	23 (64.1%)	64 (31.5%)	**<0.001**
**Others**	-	2 (5.1%)	15 (7.2%)	0.845
**HIV markers**				
**Nadir CD4+ T-cells**	-	215 (107; 343)	172 (87; 252)	**0.033**
**Nadir CD4+ T-cells<200 cells/mm³**	-	14 (38.9%)	113 (58.9%)	**0.026**
**CD4+ T-cells** #	-	832 (685; 1036)	558 (394; 808)	**<0.001**
**CD4+ T-cells<500 cells/mm³**	-	0 (0%)	83 (40.3%)	**<0.001**
**HIV-RNA >50 cp/mL**	-	0 (0%)	0 (0%)	0.999
**HCV markers**				
**HCV genotype**				
**1**	-	-	149 (72.7%)	-
**2**	-	-	5 (2.4%)	-
**3**	-	-	35 (16.5%)	-
**4**	-	-	17 (8.3%)	-
**Log10 HCV-RNA (IU/ml)**	-	-	6.29 (5.83; 6.71)	-
**HCV-RNA > 850,000 IU/ml**	-	-	144 (69.9%)	-

Statistics: Categorical variables are expressed as the absolute count (percentage). Continuous variables are expressed as the median (percentile 25; percentile 75). P-values were estimated with a nonparametric Mann-Whitney U test for continuous variables and Chi-square for categorical variables.

Abbreviations: HCV, Hepatitis C virus; HCV-RNA, HCV plasma viral load; HIV-1, Human immunodeficiency virus type 1; HIV-RNA, HIV plasma viral load; IVDU, intravenous drug user; NNRTI, non-nucleoside analogue HIV reverse transcriptase inhibitor; NRTI, nucleoside analogue HIV reverse transcriptase inhibitor; PI, protease inhibitor; II, integrase inhibitor.
